# Higher milk consumption is associated with a lower risk of diabetes mellitus: A case-control study

**DOI:** 10.1371/journal.pone.0289762

**Published:** 2023-08-16

**Authors:** Pornthep Tanpowpong, Wichai Aekplakorn, Suwat Chariyalertsak, Pattapong Kessomboon, Sawitri Assanangkornchai, Surasak Taneepanichskul, Nareemarn Neelapaichit

**Affiliations:** 1 Department of Pediatrics, Faculty of Medicine Ramathibodi Hospital, Mahidol University, Bangkok, Thailandand; 2 Department of Community Medicine, Faculty of Medicine Ramathibodi Hospital, Mahidol University, Bangkok, Thailand; 3 Faculty of Public Health, Chiang Mai University, Chiang Mai, Thailand; 4 Faculty of Medicine, Khon Kaen University, Khon Kaen, Thailand; 5 Epidemiology Unit, Faculty of Medicine, Prince of Songkla University, Songkhla, Thailand; 6 College of Public Health Sciences, Chulalongkorn University, Bangkok, Thailand; 7 Ramathibodi School of Nursing, Faculty of Medicine, Ramathibodi Hospital, Mahidol University, Bangkok, Thailand; Sohag University Faculty of Medicine, EGYPT

## Abstract

**Background & aims:**

Studies have determined that people with genetically defined lactase non-persistence have lower dairy intake that may lead to an increase risk of various non-communicable diseases. Furthermore, lactase non-persistence itself has been associated with insulin resistance. However, data on lactase non-persistence status and dairy intake in developing countries are sparse. We therefore aimed to define 1) the prevalence of lactase non-persistence among individuals with diabetes and non-diabetes in Thai population and 2) the links between lactase non-persistence, milk consumption, and risk of diabetes mellitus.

**Methods:**

We conducted a case-control study from participants of the National Health Examination Survey. DNA was isolated from the blood for LCT −13910C>T (rs4988235) polymorphism and processed using the Bio-rad c1000 touch thermal cycler and MALDI-TOF Mass Spectrometry MassARRAY Typer v4.0 (Agena Bioscience, San Diego, CA, USA) at the Center for Medical Genomics, Faculty of Medicine Ramathibodi Hospital. Cases were participants with previously diagnosed diabetes mellitus or fasting plasma glucose ≥126 mg/dL (n = 1,756) vs. the controls (n = 2,380).

**Results:**

We included 4,136 participants, 62% female, and 98.8% were > 30 years old. Homozygous CC genotype (i.e., lactase non-persistence) was noted in 98.6% and only 1.4% carried heterozygous CT. Most (76%) consumed milk <1 portion/month. Participants with either CC or CT genotype had comparable milk consumption and the risk of diabetes mellitus. Males, older adults, and lower education had a lower chance of consuming milk at least one portion per month. Besides various baseline variables, we found that higher milk consumption was associated with a lower DM risk (*P* = .01).

**Conclusion:**

The prevalence of lactase non-persistence in Thai population is very high. A significant difference in milk consumption frequency in relation to the lactase non-persistence status was not found. However, higher milk consumption is associated with a lower risk of diabetes mellitus.

## Introduction

Lactose, found in the mammalian milk such as human milk or cow’s milk, is one of the most commonly consumed disaccharides in humans. The digestive and absorptive capacity of lactose depends on the proper gene expression of lactase-phlorizin hydrolase (*LCT*), also known as ‘lactase’ enzyme, at the enterocytes in conjunction with an intact small intestinal mucosa [1].

Enattah and colleagues reported that a DNA variant, C/T-13910 from the *LCT* locus was associated with biochemically verified lactase non-persistence (LNP) in the Finnish families [2], this mutation has then become a major factor responsible for maintaining the persistence of *LCT* gene expression. Individuals carrying homozygous CC genotype are classified as LNP and the ones who carry CT or TT genotype are considered as lactase persistence (LP) [3]. A recent meta-analysis demonstrated that the global prevalence of lactose maldigestion is 68% with large ethnic differences, with the most prevalent in the South Asian and South East Asian background and the least prevalent in the Scandinavians [4]. When analyzing only the studies involving the genotype, the prevalence became 74%. Unfortunately, the previously cited studies from Thailand defined lactose malabsorption via hydrogen breath test (one study, 39 adults) [5] and lactose tolerance test (one study, 140 children) [6]. To our knowledge, no available data on genetic testing of LNP/LP status have been reported in the Thai population; therefore the prevalence of LNP remains undefined.

Studies have determined that people with LNP are more likely to consume lower intake of dairy products as compared to individuals with LP [7, 8]. Countries with higher prevalence of LNP also consume less amount of dairy products as compared to the countries with lower LNP prevalence [9]. Hypothetically, the LNP people may be more likely to suffer from intolerant symptoms after ingesting lactose-containing products. Therefore, these people may regularly avoid drinking cow’s milk or eating dairy products. People who consume lower amount of milk products may be at risk for not only nutritional inadequacy [10, 11], but also various non-communicable diseases (NCDs) [12] including diabetes mellitus (DM), hypertension, metabolic syndrome and even increased risk of death [13]. Furthermore, LNP itself (regardless of milk consumption) has been shown to be at risk for having abdominal obesity [14], insulin resistance [15], and metabolic syndrome [16]. However, LNP has been shown to be a ‘protective’ effect for obesity in the people of some ethnic backgrounds [8, 17]. Furthermore, studies defining the association between LNP status and intake of dairy products are sparse in the developing countries. Our aims were therefore to define 1) the prevalence of LNP in the selected cohort and 2) the links between LNP, milk consumption and risk of DM. Knowing this data may provide a platform for further studies to investigate various health disadvantages related to inappropriate intake of dairy products and LNP and potential disease prevention strategy.

## Materials and methods

We conducted a case-control study gathered data from participants of the 6^th^ Thai National Health Examination Survey VI (NHES VI), conducted in 2020. This survey was a comprehensive large-scale multi-region national survey that recruited over 30,000 participants starting from age 1 year old; while the blood tests were performed in older children aged ≥10 years and adults. The main aim of the survey was to define the prevalence of various NCDs and their associated risk factors. All participants or participant’ caregivers provided informed consent at the initial NHES survey for the future data reporting and leftover blood testing such as DNA extraction and sequencing. The present study included all cases with DM defined as having fasting plasma glucose (FPG) of ≥126 mg/dL or history of physician diagnosed DM who taking lower-glucose level medication. Controls were a randomly selected subsample of participants without diagnosed DM and having FPG <126mg/dL. Both cases and controls were sequenced for C/T-13910 polymorphism. Written informed consent was performed in all participants aged >18 years and the caregiver’s participants in minors aged <18 years. This study was reviewed and approved by the Human Research Ethics Committee, Faculty of Medicine Ramathibodi Hospital, Mahidol University, Thailand.

Data included baseline demographic information, education level (primary school: up to 6 years of education; secondary school: 7–12 years of education, bachelor degree, and higher than bachelor degree), anthropometric measurements, activity level, documented hypertension, dyslipidemia, and metabolic syndrome. Height and weight were measured with light clothing and no shoes. Waist circumference was measured midway between the lowest rib and the iliac crest using an anthropometric tape. Trained personnel measured blood pressure with a validated semiautomatic sphygmomanometer in a seated position after a 5-min rest. Physical activity was estimated by using global physical activity questionnaire and classified as low, moderate, high activity. Metabolic syndrome was defined based on the modified National Cholesterol Education Program’s Adult Treatment Panel III as having at least 3 of the following items: waist circumference >90 cm in men or >80 cm in women (i.e., abdominal obesity), triglyceride >150 mg/dL (i.e., hypertriglyceridemia), high-density lipoprotein cholesterol <40 mg/dL in men or <50 mg/dL in women, systolic blood pressure ≥130 mmHg or diastolic blood pressure ≥85 mm Hg or taking hypertension medications, or FPG ≥100 mg/dL or taking diabetes medications [18]. The frequency of milk consumption (including whole milk, yoghurt and low-fat milk) was also objectively measured and classified as less than once a month and above. Alkaline phosphatase was measured as an indirect marker for the bone status which was considered high if the level was >140 U/L.

Serum samples were analyzed in a central laboratory in Faculty of Medicine Ramathibodi Hospital. The analyses were performed using Dimension ExL 200 analyzer (Siemens Healthcare Diagnostics, USA) and Alinity C, (Abbott Laboratories, USA). Serum glucose was analyzed using enzymatic (Hexokinase/G6-PDH. Alkaline phosphatase was analyzed using AMP buffer p-nitrophenyl phosphate. Serum triglyceride and HDL-C were analyzed using lipase/glycerol kinase/glycerol-3-phosphate oxidase and homogenous assay, respectively. The laboratory tests were standardized according to the Centers for Diseases Control and Prevention, Lipid standardization program.

### DNA extraction and genotyping

Genomic DNA was isolated from blood samples and processed at the Center for Medical Genomics, Faculty of Medicine Ramathibodi Hospital. The LCT −13910C>T (rs4988235) polymorphism was determined using the Bio-rad c1000 touch thermal cycler and MALDI-TOF Mass Spectrometry MassARRAY Typer v4.0 (Agena Bioscience, San Diego, CA, USA). Information on probes and PCR conditions for genotyped single nucleotide polymorphisms can be obtained from the authors upon request.

### Statistical analysis

We performed all analyses using Stata version16 (StataCorp, Texas). Data are expressed as number (percent, %), median (interquartile range, IQR), and proportions (with 95% confidence interval [95% CI]). Comparisons of discrete variables across different groups were assessed using χ^2^ test or Fisher exact test, if applicable. Univariate and multivariable logistic regression analyses were performed to define various factors associated with the diabetes status. The independent variables included age (10–44, 45–59, and ≥60 years), sex (male/female), body mass index (BMI) (kg/m2), abdominal obesity (yes/no), physical activity level (low, moderate, and high), hypertension (yes/no), lactase genotype (CC, CT or TT genotype) and milk consumption (≥1/month vs. < 1/month). Adjusted odds ratio (OR) and 95% confidence interval were presented. *P* value of < .05 was considered statistically significant.

## Results

This study included 4,136 participants (1,756 cases and 2,380 controls). Most (62.2%) were female and older than 30 years old (98%). Approximately two-thirds received highest education at the primary school level. The mean (SD) body mass index (BMI) was 24.1 (5.1), 52% had BMI 25–29.9, and 59% had abdominal obesity. **Table 1** demonstrated characteristics comparing between cases and controls. When compared to controls, the cases were more likely to be males, older, received lower education, had higher BMI, less active, had diagnosed hypertension and metabolic syndrome (*P* < .05). We found that 98.6% of the participants had CC genotype for *LCT*-13910C>T polymorphism (i.e., LNP) with a comparable proportion of CC/CT genotypes between cases and controls (*P* = .63). None had TT genotype. Most (76%) participants had milk consumption less often than one portion per month, and only minority had high alkaline phosphatase level (>140 U/L).

**Table 1:** Baseline data of participants in the survey in according to the diabetes mellitus status.

Variables	Total (n = 4,136)	No DM (n = 2,380)	DM (n = 1,756)	*P*
***Baseline*, *anthropometric and activity data***				
Male, n (%)	1,561 (37.8)	857 (36.0)	704 (40.1)	.007
Age group				
10–44 years, n (%)	466 (11.3)	303 (12.7)	163 (9.3)	< .001
45–59 years, n (%)	1,197 (29.0)	739 (31.1)	458 (26.1)	
>60 years, n (%)	2,473 (59.7)	1,338 (56.2)	1,135 (64.6)	
Highest education level				
-Primary school or lower, n (%)	2,704 (65.8)	1,449 (60.9)	1,255 (71.5)	< .001
Height, cm, mean (SD)	157.8 (9.2)	157.9 (9.3)	157.0 (8.5)	.97
BMI, mean (SD)	24.1 (5.1)	23.9 (5.1)	26.3 (5.0)	< .001
BMI of 25–29.9, n (%)	2,142 (52.0)	1,183 (49.7)	959 (54.7)	.003
Abdominal obesity, n (%)	2,422 (58.9)	1,306 (55.3)	1,116 (63.7)	< .001
BMI of 25–29.9/ abdominal obesity, n (%)	2,685 (65.2)	1,471 (62.2)	1,214 (69.3)	< .001
Activity level, n (%)				< .001
Low	1,504 (36.4)	820 (34.5)	684 (39.0)	
Moderately active	946 (22.9)	521 (21.9)	425 (24.2)	
Highly active	1,686 (40.8)	1,039 (43.7)	647 (36.9)	
***Non-communicable diseases***				
Hypertension, n (%)	1,844 (48.2)	899 (40.5)	945 (58.7)	< .001
Cholesterol level				
≤200 mg/dL, n (%)	1,662 (40.2)	788 (33.1)	874 (49.8)	< .001
>200 mg/dL, n (%)	2,473 (59.8)	1,592 (66.9)	881 (50.2)	
Metabolic syndrome, n (%) (N = 4,100)	1,980 (48.3)	701 (29.7)	1,279 (73.3)	< .001
***Lactase gene and milk consumption***				
13910C>T polymorphism				
CC genotype (lactase non-persistence)	4,079 (98.6)	2,349 (98.7)	1,730 (98.5)	.63
CT genotype (lactase persistence)	57 (1.4)	31 (1.3)	26 (1.5)	
Frequency of milk consumption (n = 4,127)				
< 1/month, n (%)	3,142 (76.1)	1,758 (74.1)	1,384 (78.9)	< .001
≥ 1/month, n (%)	985 (23.9)	615 (25.9)	370 (21.1)	
Alkaline phosphatase >140 U/L, n (%)	150 (3.6)	57 (2.4)	93 (5.3)	< .001

**Abbreviations:** BMI, body mass index; DM, diabetes mellitus.

**Table 2** demonstrated factors associated with higher milk consumption (≥ 1/month). Males, older adults (>60 years), participants with lower education and individuals with metabolic syndrome were less likely to have consuming milk ≥1 portion/month. Only 21.2% in adults >60 years consumed milk ≥ 1 portion/month *vs*. 34.3% among the ones aged <45 years. We found that 23.8% of CC individuals consumed milk ≥1 portion/month *vs*. 28.1% among the 57 CT individuals. The milk consumption frequency in according to the CC/CT genotypes was comparable. We found that 44.8% of participants who consumed milk ≥1 portion/month had metabolic syndrome compared to 49.4% among the ones who consumed less milk products (*P* = .06). Proportions of metabolic syndrome were comparable between the two *LCT* genotypes (*P* = .76).

**Table 2:** Factors associated with higher milk consumption (≥ 1/month).

Variables	Odds ratio	95% CI	P
Male	0.83	0.71, 0.97	.017
Age group (reference group: 10–44 years)			
45–59 years	0.85	0.67, 1.08	.18
>60 years	0.76	0.61, 0.97	.03
Education level	0.54	0.46, 0.64	< .001
Primary school (reference group: higher education)			
Metabolic syndrome	0.87	0.75, 1.009	.067
CC (vs. CT) genotype	1.15	0.64, 2.07	.65
Alkaline phosphatase (> 140 vs. < 140 U/L)	1.03	0.69, 1.54	.89

Independent risk factors of DM included male sex, lower education level, abdominal obesity and associated hypertension, while the protective factors were high activity level and a higher frequency of milk consumption (**Table 3**).

**Table 3:** Risk factors of diabetes (known diabetes and/or fasting plasma glucose ≥126 mg/dL) (N = 4,083).

Variables	Odds ratio	95% CI	P
Male	1.36	1.18, 1.56	< .001
Age group (reference group: 10–44 years)			
45–59 years	0.91	0.72, 1.16	.46
≥60 years	0.93	0.73, 1.18	.57
Highest education level (primary school or less)	1.46	1.25, 1.69	< .001
Body mass index	1.01	0.99, 1.03	.29
Abdominal obesity	1.40	1.18, 1.67	< .001
Activity level (reference group: low activity)			
Moderately active	0.96	0.81, 1.14	.66
Highly active	0.77	0.67, 0.90	.001
Hypertension	1.89	1.65, 2.17	< .001
CC (vs. CT) genotype	1.32	0.76, 2.27	.32
Milk consumption (≥1/month vs. < 1/month)	0.82	0.71, 0.96	.01

## Discussion

We found a very high prevalence (98.6%) of CC genotype which is defined as LNP in both cases and controls. A recent systematic review in 2017 demonstrated that Thai population had an 84% prevalence of lactose maldigestion defined by either hydrogen breath test or lactose tolerance test but not the formal genetic testing for LNP/LP polymorphism [4]. Our primary aim of this study was to define the prevalence of LNP gene but not the status of lactose maldigestion. Many individuals suffer from symptoms of lactose intolerance but did not have documented lactose maldigestion or the intolerant ones may carry the lactase persistence gene [19]. Therefore, the LNP status may not necessarily predict the amount of milk consumption in the population, for example, individuals with the CC genotype may not avoid milk consumption as they may not have intolerant symptoms during the surveyed period as their *LCT* gene expression may remain well-functioned. The ones with CC (LNP) or CT (considerate LP) genotype may have their gene expression down-regulated at a different age [1].

A relatively small proportion (24%) of the study population consumed milk products at least once a month. We believed that most participants in our cohort consumed small amount of milk intake due to some plausible reasons: 1) dairy products are not the main dietary source in the country especially among older adults as compared to other sources of protein such as hen’s egg, pork or chicken which are the primary sources of animal protein locally; and 2) participants had a relatively homogenous genetic mutation (predominately CC genotype) that may lead to gastrointestinal symptoms of lactose maldigestion such as abdominal pain, distension, bloating, flatulence or watery diarrhea at any time so they may limit their milk intake due to the perceived symptoms [20]. We however could not demonstrate a significant different in the frequency of milk consumption between participants with CC vs. CT (Table 2), similar to a study by Corella et al. [8]. In contrast to our finding, various studies have shown that LNP is associated with lower milk consumption in some population [21, 22]. We also attempted to measure alkaline phosphatase which is an indirect bone marker and the elevated level may suggest suboptimal bone health, hence we found only 3.6% who had higher alkaline phosphatase (>140 U/L) which did not differ between the two *LCT* genotypes. However, a higher proportion of DM patients had high alkaline phosphatase (>140 U/L) when compared to the non-DM counterparts which could be explained by several reasons such as more obese patients and lower milk consumption in the DM group. Obesity is a well-known risk factor of vitamin D deficiency which can cause an elevation of alkaline phosphatase. Non-alcoholic fatty liver disease itself usually causes an elevation of liver transaminases but not the alkaline phosphatase. Males, older participants, and the ones who received lower education had less frequent milk intake (Table 2). Sex-related difference on milk consumption is difficult to explain by biologic plausibility but may potentially be explained by social construct. Older adults may perceive that they do not need to consume milk products as compared to the young adults. The older people with an at-risk genotype of LNP may also have a higher chance that they would develop symptoms after ingesting milk or dairy products when they get older.

A trend of higher milk consumption was associated with a lower chance of metabolic syndrome (*P* = .06), the finding that is consistent with the recent systematic review and meta-analysis [23]. Long-term dairy supplementation showed a weak anti-inflammatory effect in both the healthy adults and also obese adults or the ones with metabolic syndrome [24]. Reverse causality could also explain this association too as patients with already known or previously diagnosed metabolic syndrome may drink less cow’s milk or consume less dairy-containing products. On the other hand, *LCT* genotypes had no effect on metabolic syndrome (*P* = .90, data not shown), similar to a study by Corella et al. [8]. On the other hand, a study from Brazil included 334 adults from European descent noted that adults with CC genotype had a higher risk of metabolic syndrome. Interestingly, our study had no participants with TT genotype which may explain the different proportion of metabolic syndrome related to the *LCT* gene status when compared to the previous studies from westernized countries [16].

Male sex, lower education level, abdominal obesity, and hypertension were associated with DM as shown in Table 3. A study performing in mice found that perinatal high-fat diet in the mothers increases the risk of impaired glucose tolerance only in the male offspring, not the females. These male mice also had increased body weight and adipose tissue mass in relation to maternal high-fat diet [25]. Obesity is also a well-known risk factor of diabetes [26]. We found that higher milk intake was associated a lower chance of DM. Evidence has shown that higher dairy consumption has been linked to a lower risk of type 2 DM via various protective roles of dairy protein. Bioactive peptides and amino acids from milk proteins delayed gastric emptying, enhanced incretin and insulin responses therefore lowering glucose level especially during the postprandial period [27]. However, a Mendelian randomization study from Denmark in 2015 showed that higher milk intake was not associated with a lower risk of type 2 DM or overweight-obesity either by observationally or genetically via LNP/LP status [28]. Both *LCT* genotypes had comparable proportions of DM. Similar to our study, Pienar et al. also found that 87 children aged 6–17 years (52% with LNP gene) had comparable FPG level between the LNP and LP individuals [29].

### Strengths and limitations

From a case-control study selected from a large population-based national survey, we herein report a prevalence of genetically proven LNP/LP status among both diabetes and without diabetes in the Thai population for the first time. However, we unexpectedly found a very small number of subjects with CT genotype and none with TT genotype which limited analyses on the interested variables related to the LNP/LP status. The survey consisted of detailed information from the food frequency questionnaire. The nature of case-control study limits the ability to define disease prevalence of the population, however, the control group was relatively representative of the Thai population and found the similar prevalence of LNP of both groups. This might suggest that the prevalence in the general population maybe somewhat in between that of cases and controls. Lastly, if data on the symptoms of lactose maldigestion/intolerance can be gathered with additional tests on bone markers such as calcium and vitamin D status, we should be able to better explain the link between LNP, milk consumption, and various NCDs.

## Conclusions

The prevalence of LNP as defined by CC genotype in the Thai population is very high with a small proportion with CT genotype (i.e., LP population). We could not demonstrate a significant difference in the frequency of milk consumption, the risk of DM in relation to the LNP/LP genotypes. However, higher milk consumption demonstrated a tendency that it may attenuate the risk of metabolic syndrome and hyperglycemia. Further population-based studies on milk consumption associated with the risks of subsequent hyperglycemia or DM are required.

## Supporting information

S1 Table Baseline data of participants in the survey in according to higher milk consumption (≥ 1/month) status. (DOCX)

## Abbreviations

DM: diabetes mellitus
FPG: fasting plasma glucose
LNP: lactase non-persistence
LP: lactase persistence
NCD: non-communicable disease
