# Supplementary material for: Higher milk consumption is associated with a lower risk of diabetes mellitus: A case-control study
Source: PLoS One. 2023 Aug 16;18(8):e0289762. doi: 10.1371/journal.pone.0289762 (PMC10431601; doi:10.1371/journal.pone.0289762)
Supplement: S1 Table — (DOCX) [file pone.0289762.s001.docx]

**Supporting information**

**Table S** Baseline data of participants in the survey in according to higher milk consumption (≥ 1/month) status

| **Variables** | **Milk consumption <1/month (n= 3,142)** | **Milk consumption ≥ 1/month (n=985)** | ***P-value*** |
| --- | --- | --- | --- |
| Male, n (%) | 1,213 (38.6) | 345 (35.0) | 0.04 |
| Age group |  |  |  |
| 10-44 years, n (%) | 305 (9.7) | 159 (16.1) | <.001 |
| 45-59 years, n (%) | 895 (28.5) | 300 (30.5) |  |
| >60 years, n (%) | 1,942 (61.8) | 526 (53.4) |  |
| Highest education level  -Primary school or lower, n (%) | 2,172 (69.5) | 527 (53.8) | <.001 |
| Height, cm, mean (SD) | 157.0 (8.4) | 157.3 (8.2) | 0.45 |
| BMI, mean (SD) | 25.9 (4.8) | 25.6 (4.7) | 0.23 |
| BMI of 25-29.9, n (%) | 1,643 (52.5) | 496 (50.5) | 0.27 |
| Abdominal obesity, n (%) | 1,844 (59.0) | 574 (58.5) | <0.76 |
| BMI of 25-29.9/ abdominal obesity, n (%) | 2,041 (65.3) | 640 (65.2) | <0.96 |
| Activity level, n (%) |  |  | <0.001 |
| Low | 1,134 (36.1) | 368 (37.4) | 0.44 |
| Moderately active | 731 (23.4) | 210 (21.3) |  |
| Highly active | 1,277 (40.6) | 407 (41.3) |  |
| ***Non-communicable diseases*** |  |  |  |
| Hypertension, n (%) | 1,450 (49.5) | 392 (43.8) | <0.01 |
| Diabetes, n (%). | 1384 (44.1) | 370 (37.6) | <0.001 |
| Cholesterol level |  |  |  |
| ≤200 mg/dL, n (%) | 1,281 (40.8) | 379 (38.5) | 0.20 |
| >200 mg/dL, n (%) | 1,860 (59.2) | 606 (61.5) |  |
| Metabolic syndrome, n (%) (N = 4,100) | 1,541 (49.4) | 437 (44.8) | 0.01 |
| ***Lactase gene and milk consumption*** |  |  |  |
| 13910C>T polymorphism  CC genotype (lactase non-persistence)  CT genotype (lactase persistence) | 3,101 (98.7)  41 (1.3) | 969 (98.4)  16 (1.6) | 0.45 |
| Alkaline phosphatase >140 U/L, n (%) | 115 (3.7) | 34 (3.4) | 0.76 |

**Abbreviations:** BMI, body mass index
